# Fast max-margin clustering for unsupervised word sense disambiguation in biomedical texts

**DOI:** 10.1186/1471-2105-10-S3-S4

**Published:** 2009-03-19

**Authors:** Weisi Duan, Min Song, Alexander Yates

**Affiliations:** 1 Department of Computer and Information Sciences, Temple University, Philadelphia, PA 19122, USA; 2 Information Systems Department, New Jersey Institute of Technology, Newark, NJ 07102, USA

## Abstract

**Background:**

We aim to solve the problem of determining word senses for ambiguous biomedical terms with minimal human effort.

**Methods:**

We build a fully automated system for Word Sense Disambiguation by designing a system that does not require manually-constructed external resources or manually-labeled training examples except for a single ambiguous word. The system uses a novel and efficient graph-based algorithm to cluster words into groups that have the same meaning. Our algorithm follows the principle of finding a maximum margin between clusters, determining a split of the data that maximizes the minimum distance between pairs of data points belonging to two different clusters.

**Results:**

On a test set of 21 ambiguous keywords from PubMed abstracts, our system has an average accuracy of 78%, outperforming a state-of-the-art unsupervised system by 2% and a baseline technique by 23%. On a standard data set from the National Library of Medicine, our system outperforms the baseline by 6% and comes within 5% of the accuracy of a supervised system.

**Conclusion:**

Our system is a novel, state-of-the-art technique for efficiently finding word sense clusters, and does not require training data or human effort for each new word to be disambiguated.

## Background

Word sense disambiguation is a central problem for biomedical text mining. A variety of applications depend on it, such as biomedical information extraction [1] and literature discovery [2,3]. Certain kinds of terms are prone to being especially ambiguous, such as the names of proteins and genes, and the literature is replete with acronyms that have multiple meanings. While humans find it relatively easy to determine the correct sense of a word given its context, so far automatic approaches have not met the same kind of success.

Existing techniques include supervised systems and knowledge-based systems, which both require extensive manual effort per ambiguous term in order to build an accurate system. Supervised systems require multiple examples of each sense of a word in context, manually labeled with the correct sense.

From this data, a supervised system can learn to predict the correct sense of the same word in a new context. However, these data sets are labor-intensive, time-consuming, and expensive to produce, and thus far have been produced for only several dozen ambiguous terms. It is impractical to scale this kind of technique to all terms used in the biomedical literature.

Knowledge-based systems use databases and dictionaries to disambiguate words. For example, Schijvenaars *et al*. [4] compare the words used in the definitions of gene symbols with the terms surrounding a gene symbol in an abstract to disambiguate the symbol. As with supervised systems, knowledge-based systems require extensive manual effort per ambiguous term in order to create the databases and dictionaries. In practice, far more terms are included in widely available databases than in supervised datasets. However, new terms and new usages of existing terms are constantly being invented, and even databases like the Unified Medical Language System (UMLS) [5] do not cover all senses of all terms in the biomedical literature. Thus the knowledge-based techniques have also not scaled to every possible ambiguous term.

In response to this situation, we have developed a novel system for disambiguating biomedical terms. Our system, called SENSATIONAL, has only a single free parameter, and it can be trained on only a few hundred manually-labeled examples of one ambiguous term in context. Following previous work in Natural Language Processing (*e.g*., [6,7]), we call this system unsupervised despite its use of training data. This is because in contrast to existing work, it has a very small number of parameters (in our case, just one), which are essentially thresholds that could be chosen judiciously by hand, but may also be set with a simple training procedure. Furthermore, the parameter is not tied to the vocabulary, so the system does not need to be retrained when ported to a new document collection. After being trained the system can be applied to any new term. SENSATIONAL takes as input a set of text documents or sections of documents that contain an ambiguous term, and clusters the documents into groups representing the different senses of the term.

To be effective, SENSATIONAL must be both accurate and fast, so that it can handle large document collections with many ambiguous terms. Its clustering algorithm is a promising new maximum-margin technique, and makes the following contributions to the state of the art:

1. SENSATIONAL **provides state of the art accuracy in comparison with other unsupervised techniques, and approaches the accuracy of supervised techniques**. On one standard data set for biomedical word sense disambiguation developed by the National Institute of Standards and Technology (NIST), SENSATIONAL achieves a precision of 0.61, within five points of Leroy and Rindflesch's supervised system [8]. This is an impressive achievement, given that the supervised techniques have access to a number of manually-labeled examples for each term in the data set to learn from. On an extended data set that includes a variety of acronyms, SENSATIONAL achieves a precision of 0.76, significantly better than a baseline system and outperforming Kulkarni and Pedersen's unsupervised SenseClusters system [9,10].

2. SENSATIONAL **can cluster *N *text segments in time *O*(*N *log *N*), making it scalable to large text collections**. Max-margin clustering is typically an expensive and slow operation, but SENSATIONAL uses an algorithm based on minimum spanning trees to find approximate solutions in the max-margin framework, while remaining efficient and accurate.

In the next section, we outline background information and previous work in biomedical word sense disambiguation. In Section we present SENSATIONAL's novel algorithm for clustering word senses. In Section we discuss a critical extension of the algorithm that makes it robust to data sparsity and outliers and dramatically improves our results. Section describes our experiments and results, and Section concludes.

## Previous work

In this section we will review Word Sense Disambiguation (WSD) techniques proposed in the biomedical domain. Word sense disambiguation is a challenging task in many areas of automatic language processing, such as machine translation [11], information retrieval [12,13], and question answering [14]. Word sense disambiguation algorithms that have been used in the biomedical domain [15-18] in three different categories: supervised learning, unsupervised learning, and knowledge-based systems.

A majority of WSD techniques is based on supervised Machine Learning (ML). Hatzivassiloglou et al. [1] used three ML techniques, namely Naïve Bayesian learning, decision trees and inductive rule training for protein, gene and mRNA disambiguation. Ginter et al. [19] introduced a statistical classification method and a weighted bag-of-words representation. They weighed the context words to assign words located closer to the ambiguous word higher weights and reported the SVM-based classification performance in gene/protein name disambiguation was improved from 79% to 82% accuracy. Pahikkala et al. [20] analyzed the kernel function and constructed smoothed word position-sensitive and smoothed word position and distance sensitive representations of the training data using kernel density estimation techniques. They demonstrated with the Senseval-3 data that the kernel improved the classification performance of SVMs compared to the ordinary Bag-of-Words kernel.

Liu *et al*. [21] compared the Naïve Bayes and their hybrid supervised WSD technique combining a Naïve Bayes and an exemplar-based algorithm on a subset of the NLM-WSD data set. They reported that their hybrid supervised technique with Naïve Bayes performed the best in the biomedical domain. Their evaluation results indicated that two feature sets containing 1) all words within a window size of three and their orientation, and 2) the three nearest two-word collocations performed best on the NLM-WSD subset. Joshi *et al*. (2005) [22] compared four different supervised ML algorithms: Naïve Bayes, Support Vector Machines (SVM), AdaBoost and Decision Trees on a subset of the NLM-WSD data set. They reported that SVM obtained the best overall accuracy. They compared unigram and bigram from the words surrounding the target word at the sentence level.

Mohammad and Pederson [23] investigated how lexical features and syntactic features contribute to WSD. The results showed that simple lexical features such as words in context and collocation used in conjunction with part of speech information achieved better results. The results also showed that certain pairs of features were redundant and others complementary in which is important to determine what features to use. Leroy and Rindflesch [24] proposed a supervised WSD system that maps words to their appropriate sense (concept) in the UMLS. They split the relations into two sets, core relations and non-core relations due to their hierarchical nature. The core relations are hierarchical such as is-a, conceptual-part-of, and consists-of. All other relations are identified as non-core relations. They also used the POS of the target word and whether the target word is a head word.

In the second category, a number of knowledge-dependent unsupervised approaches have been developed for word sense disambiguation in general English (e.g., [10,25-27]). In these studies the final clusters are evaluated either by comparing them to sense distinctions made in a general English dictionary, or by evaluating how well the method distinguishes between unambiguous words that were conflated together as a pseudo-word. Bhattacharya, Getoor, and Bengio [28] introduced two knowledge-enhanced unsupervised WSD systems: "Sense Model" and "Concept Model". They built probabilistic models using parallel corpus with an unsupervised approach. They demonstrated that the concept model improved performance on the word sense disambiguation task over the previous approaches participated in 21 Senseval-2 English All Words competition. Savova *et al*. [29] extended existing methods of unsupervised word sense discrimination to biomedical text. It adopts the experimental framework proposed by Purandare and Pedersen [10]. They created three systems that follow Schtze [26] and used second order co-occurrences as the main source of information. They showed that the method of clustering second order contexts in similarity space is effective in the biomedical domain.

The third category of WSD techniques uses established knowledge from curated terminology systems. Wren *et al*. [30] presented a collection of four databases that contain a vast list of abbreviations along with their meaning. Schijvenaars* et al*. [4] and Pahikkala *et al*. [20] developed thesaurus-based approaches to resolve gene/protein symbols. Schijvenaars *et al*. achieved 92.5% accuracy on human gene symbols. They compared a genes definition compiled from a database to abstract where the gene symbol occurs. Both definition and abstract are represented as concept finger prints, i.e., vectors of biomedical terms. Both vectors are compared by a similarity measure based on cosine. Pahikkala *et al*. followed a similar approach with Schijvenaars *et al*. But instead of using the full abstract, they defined the context of a gene symbol as a number of words before and after and achieved 85% accuracy. The experiment results showed that small datasets and clear or fuzzy borderline between senses impact on the classification task.

Liu *et al*. [21] used UMLS as the ontology and identified UMLS concepts in abstracts and analyzed the co-occurrence of these terms with the term to be disambiguated. They achieved a precision of 93% and a recall of 47%. Gaudan *et al*. [31] used SVMs on their algorithm to resolve abbreviations in MEDLINE and obtained a precision of 98.9% and a recall of 98.2%. They simplified the disambiguation task by excluding rare senses (appearing in less than 40 documents) from the test set and keeping in the training set only the ambiguous short-forms that also had long-forms in the documents. Humphrey *et al*. [32] adopted the Journal Descriptor Indexing (JDI) methodology to tackle the ambiguity problem to map free text to terms from the UMLS metathesaurus. JDI combines a statistical, corpus-based method with utilization of pre-existing medical domain knowledge sources. They used the 45 ambiguities, and achieved that the overall average precision of the highest-scoring JDI method was 78.7% compared to 25% for their baseline method based on the frequency counts of MeSH terms in a document subset.

## Fast max-margin clustering

Our system is built on the principle of margin-maximization: that is, in order to find clusters in the data, it attempts to find a surface in the space of the data points that separates the points in such a way that it maximizes the smallest distance between points on opposite sides of the surface. The minimum distance that separates the two clusters is known as the *margin*. This principle has a long history in machine learning theory and in practice in the form of Support Vector Machines [33]. Our clustering algorithm uses this criterion to efficiently decide among the vast number of possible clusterings of the data, and experiments described in the next section validate this approach.

Exact max-margin clustering has been shown in recent years to be a highly accurate and effective technique, but it is computationally expensive. Approximate solutions, such as a reduction to linear programming [34] or the use of support vector regression and optimization techniques for quadratic programming [35], make it more practical, but so far have not made it scale to large data sets.

We use a novel approximation algorithm for max-margin clustering based on minimum spanning trees, a graph data structure that can be computed efficiently. We first briefly review the concept of minimum spanning trees and describe how they can be used for clustering. We then provide a detailed algorithm, a performance analysis, and a set of theoretical guarantees.

### Minimum spanning trees for clustering

A spanning tree of an undirected graph is a subgraph that contains all of the nodes of the original graph, but only enough edges so that every node has exactly one path to every other node. Naturally, if the original graph is disconnected, the spanning tree will be as well. In general, spanning trees are not unique for a graph. For a weighted graph *G*, let the score of a spanning tree be the sum of the weights on all edges in the tree. A minimum spanning tree (MST) for *G *is a spanning tree such that its score is less than or equal to the score of all other spanning trees for *G*. MSTs also need not be unique in general. Given a weighted, undirected graph *G *with vertices *V *and edges *E*, the well-known Prim's algorithm can find a minimum spanning tree of the graph in time *O*(*E *+ *V *log *V*) when implemented with Fibonacci heaps [36].

Minimum spanning trees have an important property that makes them useful for a margin-maximization problem. Let *T *be a minimum spanning tree of *G*, and consider the edge *e *of *T *with the largest weight of any edge in *T*. *e *separates two parts of the tree by a distance equal to its weight. If we remove *e *from *T*, *T *will be separated into two components with no edge connecting them. The margin that separates these two components is exactly equal to the weight of *e*, since *e *is the smallest edge in the graph *G *that connects them – by definition of the minimum spanning tree, if there were a smaller edge than *e *that connected the two components, it would have been part of the MST to begin with. Thus, the largest edge of *T *provides a large margin between two components of G. This insight forms the basis of our algorithm.

### Detailed clustering algorithm

The input to our algorithm consists of the ambiguous term of interest and a set of documents containing that term. The objective is to group all mentions of the ambiguous term in the documents into clusters such that members of the same cluster have the same meaning, and a different meaning from that of other clusters. Note that unlike the bulk of previous work, the algorithm assumes essentially no inputs that require significant manual input to construct, such as manually-labeled training examples for supervised learning algorithms or manually-constructed and manually-curated databases containing structured knowledge. This key attribute of the algorithm makes it applicable to any ambiguous term for which there are a significant number of mentions in text (on average 271 in our experiments), and thus applicable in settings like information extraction or information retrieval where the user or application might be interested in arbitrary terms.

Like almost any clustering algorithm, our algorithm requires a representation of the data in a feature space and a distance function that indicates how far apart two data points are. We use the well-known bag-of-words representation for the mentions of the ambiguous terms, with a stop-word list of approximately 100 very common tokens. After experimentation with a variety of distance functions, we determined that the following function of two feature vectors provides an efficient and effective measure of the distance between them:

(1) $$d({\hat{v}}_{1},{\hat{v}}_{2})=\frac{\sum_{i}\left(v_{1}^{i}+v_{2}^{i}\right)}{\sum_{i}\min(v_{1}^{i},v_{2}^{i})}$$

In principle, our algorithm will work with any feature representation or distance function.

**Figure 1:**
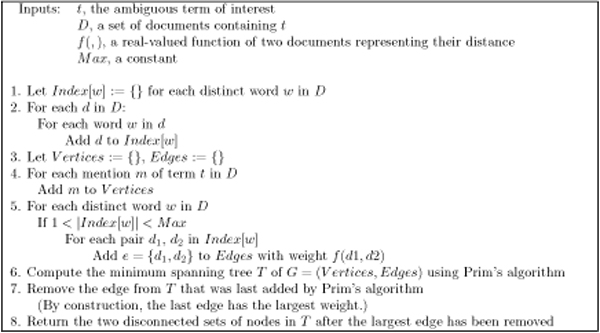
**SENSATIONAL clustering algorithm**. SENSATIONAL's clustering algorithm is a fast, approximate technique for finding maximum-margin clusters in document collections. It uses minimum spanning trees to find high-weight edges connecting two components of the graph.

Our algorithm appears in Figure 1. The first five steps build a graph that represents the set of mentions of an ambiguous term, and how far apart each mention is from every other one. Steps six through eight construct an MST for the graph, and cluster the vertices of the graph using the MST.

The first part of this process creates a data structure, the *Index*, that stores for each word a set of documents containing that word. This index can be used to prune the set of edges that are added to the eventual graph: after step five, the set of edges contains only those pairs of documents that share at least one word in common (because they both appeared in the same set *Index*[*w*] for some *w*). Furthermore, the word that they shared must not have appeared in more than *Max *documents. This restriction prevents the comparison of documents if they share only function words like "the" or other relatively uninformative words that appear in almost every document. This algorithm has previously been shown to limit the number of comparisons made between documents to *O*(|*V*| log |*V*|) [37]. Limiting the number of comparisons between documents in turn limits the number of edges in the graph *G*, making further processing more efficient. In practice, we set *Max *to 20.

Once the graph *G *is constructed, the algorithm builds a minimum spanning tree using Prim's algorithm, and removes the largest edge from it. Since a MST contains exactly one path between every pair of nodes, the act of removing the largest edge must remove every path between nodes on opposite sides of the edge. In other words, the MST without its largest edge contains two disconnected components. The algorithm returns these two components as the clusters. Note that the weight of the largest edge of the MST is the margin between the two clusters, since by definition of the MST there can be no shorter edge that connects the two clusters. By removing the largest edge of the MST, the algorithm finds a clustering that forces a large-margin separation between the resulting disconnected components.

### Analysis and performance guarantees

Maximum-margin clustering is a computationally challenging task, especially for large document collections. Xu *et al*. [34] show that it is equivalent to convex integer programming, which is NP-hard. Our algorithm gives an approximate answer in a highly efficient manner, making it applicable to large document collections. It offers the following guarantees:

1. The margin of the clustering is exactly equal to the weight of the largest edge in the MST.

2. The margin of the clustering is greater than or equal to the weight of every edge in the MST.

For a graph of *N *vertices, there are *N *-1 edges in the MST, so the margin is greater than or equal to at least *N *- 1 edges of the graph.

3. For Zipf-distributed data, the clustering can be computed in time *O*(|*D*| + *N *log *N*), where *N *is the number of mentions of the ambiguous term of interest, and *D *is the set of documents containing the term. Construction of the index takes time *O*(|*D*|). Construction of the graph takes time *O*(*N *log *N*) and produces a graph with at most *O*(*N *log *N*) edges [37]. Finally, running Prim's algorithm and computing the clustering takes time *O*(|*E*| + |*V*| log |*V*|) = *O*(*N *log *N*).

## Handling data sparsity and outliers

While the MST-based algorithm above has proved capable of finding maximum-margin clusterings in practice, one property of our data has caused those clusterings to be unusable for word sense disambiguation: outliers. In particular, document collections often have a number of documents that are very different from all other documents in the collection and share almost no words in common with any other document. Calling them outliers is indeed somewhat misleading, since there are so many of them. In fact, in one of our test collections, over 20% of the documents were outliers in the sense that they shared less than 10% of their words with the closest document in the collection. This problem is related to the notions of data sparsity and the curse of dimensionality; it plagues many natural language processing systems.

Outliers cause our MST-based algorithm to go seriously wrong. If we use the minimum spanning tree algorithm to cluster a document collection with a number of outliers, it consistently removes an edge that separates one of the outlier points from the rest of the collection. The algorithm is properly finding a large-margin clustering, since the outlier points are very far from every other point in the collection, so they are separated from the rest of the collection by a wide margin. However, the resulting clusters are poor results for disambiguating word senses. This problem proved to be pervasive in our initial experiments, making the accuracy of our system roughly the same or slightly worse than a baseline system in which all points are simply grouped into a single cluster (a.k.a., the All-in-1 baseline). Thus our original algorithm has terrible performance due to this one fatal flaw.

In response, we have developed an efficient mechanism for purifying our data set into a set of points that represent the core documents in the collection. We then apply our clustering technique to these core points, determine a clustering for them, and add each outlier point to the cluster of core points to which it is closest. Our experiments show that with this technique for handling outliers, the performance of SENSATIONAL's algorithm improves drastically.

Our algorithm for finding the core points of the graph operates on the minimum spanning tree produced by our clustering algorithm. It has two major steps: first, it counts the number of nodes in every sub-tree of the MST. Second, it finds a "backbone" of edges connecting nodes that are the roots of large sub-trees. The backbone, which contains only nodes that are relatively close to a number of other documents, is then used as the core of the MST. We explain each step in more detail below.

### Counting the size of subtrees

**Figure 2:**
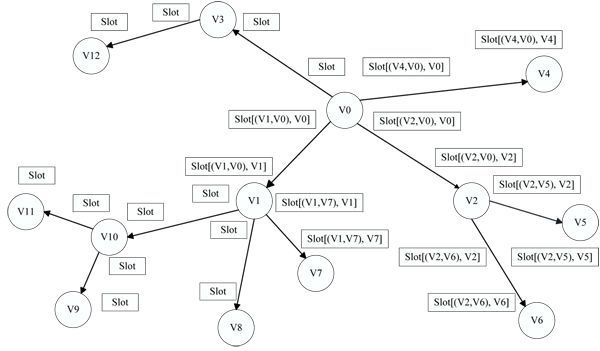
**A minimum spanning tree with slot annotations**. Our technique for handling outliers performs a breadth-first traversal of the minimum spanning tree in order to count the number of nodes in each subtrees.

The first step in the algorithm is to calculate the size of each subtree in the MST. We first define a set of *slots *to store the number of nodes in a subtree. Each slot stores the number of nodes in a subtree rooted at a node *x *and connected to the rest of the MST by edge (*x*, *y*). Figure 2 shows an example MST with attached slots. In this figure, for example, slot [(*V*1, *V*0), *V*1] is the size of the subtree that is rooted at *V*1 and is connected to the rest of the tree along edge (*V*1, *V*0). It includes nodes *V*7 through *V*11. Notice that this subtree is different from the subtree rooted at *V*1 and connected to the rest of the tree by edge (*V*1, *V*10) – that subtree includes all nodes except *V*9, *V*10, and *V*11.

The calculation of the subtree sizes begins by choosing a random vertex as the root of the MST (node *V*0 in the figure). We then perform a breadth-first traversal of the tree, adding each node to a stack with a pointer to its parent along the way. The direction of the tree traversal is indicated by directed edges in Figure 2. After all nodes have been added to the stack, it pops the stack one node at a time, and calculates the slot for that node and the edge connecting it to its parent in the breadth-first traversal. By ordering nodes in the reverse order of the tree traversal, the slots of every child node will be calculated before the slots of the parent, and the parent will be able to calculate its slot using the value of its children's slots. Specifically, if a node *x *with parent *y *is popped from the stack, the algorithm calculates:

(2) $$\text{Slot}[(x,y),x]=1+\sum_{c\in \text{Children}(x)}\text{Slot}[(c,x),c]$$

So, for example, Slot [(*V*1, *V*10), *V*1] will be calculated as the sum of the slots for [(*V*7, *V*1), *V*7], [(*V*8, *V*1), *V*8], and [(*V*0, *V*1), *V*0], plus one for *V*1 itself.

After the stack has been completely popped, one slot will have been calculated for every edge in the tree. However, there are two slots on every edge, for each endpoint of the edge. The remaining slots can easily be filled, since the two slots on each edge must add up to the total number of nodes in the tree.

### Finding the backbone

Our Backbone-Finding Algorithm takes as input all the slots from the last algorithm and outputs the set of vertices in the backbone. Let *Backbone *be initialized to an empty set. Our algorithm first finds a vertex with some slot value that is as close as possible to half the number of nodes in the graph. Such a vertex is intuitively near the center of the MST. It breaks ties randomly. It then expands the backbone by adding the root of the biggest subtree connected to the starting point, as determined by the subtree's slot value. It repeatedly adds the root of the biggest subtree connected to either end of the backbone, until all available subtrees have slot values lower than a threshold parameter *T*. During the expansion, if it reaches a new vertex whose two biggest sub-trees do not include the one where the expansion is coming from, it just clears the previous work by setting *Backbone *= {}, and starts over by setting the new vertex as the starting point for the construction of the backbone.

#### An Example

Suppose we have *T *= 2. In the tree in Figure 2, vertices *V*0 and *V*1 both have slots equal to 6 and 7 (along the edge [*V*1, *V*0]), which is close to half the total of 13 nodes. Suppose the algorithm randomly chooses *V*1 as the starting point for the backbone. It will then add *V*0 to the backbone since the value of Slot [(*V*0, *V*1), *V*0] is 7, which is greater than the size of any of *V*1's other subtrees. Next, the algorithm will add *V*2 and *V*10 to the backbone, which both have slot values equal to 3. At that point, the Backbone-Finding algorithm stops, since none of *V*2 and *V*10's children has a slot value ≥ *T*.

After finding the backbone of this MST, our complete algorithm operates by cutting the largest-weighted edge on the backbone, and then clustering all other nodes according to which part of the backbone they are connected to in the backbone. Note that this procedure accomplishes our original goal of forcing the algorithm to cut large edges that are not simply separating outliers from the rest of the group. Experiments below validate our intuitive approach.

### Algorithm analysis

As with our MST algorithm, the Backbone-Finding procedure is designed to operate efficiently so that it can be applied to large collections of documents. The calculation of slot values requires a breadth-first traversal of the tree, which takes *O*(*N*) time for *N *vertices. It then requires popping each of the nodes and adding the slot values of incoming edges. Since there are *N *- 1 edges in a tree of *N *vertices, and each edge is considered at most twice during this step, it also takes *O*(*N*) time. Calculating the leftover slots after popping the stack requires visiting each edge again, for another *O*(*N*) steps. Determining the backbone from the MST with slot values uses a simple, greedy expansion technique that visits each node at most once. In total, the whole Backbone-Finding procedure requires *O*(*N*) time.

## Experiments and results

We performed two experiments to test the accuracy of our SENSATIONAL system against both unsupervised and supervised systems.

### Experimental setup

We tested SENSATIONAL on two data sets. To compare SENSATIONAL with a supervised system, we tested it on a standard data set of ambiguous biomedical terms available from the National Library of Medicine (NLM) [3]. Leroy and Rindflesch [8] have developed a system using a Naïve Bayes classifer on this same data set, and we show the results for both systems below. This data set contains 100 examples of each term being used in context. Each example is labeled with its sense in the Unified Medical Language System (UMLS) dictionary of word senses, or with the label "None" if no corresponding sense is found in UMLS. Following Leroy *et al*., we evaluate on the subset of 15 terms for which the majority sense makes up at most 70% of the examples; this way, there is a reasonable amount of data for the system to learn patterns identifying the minority sense(s).

The NLM data set contains a variety of biomedical terms, but it leaves out an important type of ambiguous keyword: acronyms. We further evaluated SENSATIONAL on a data set of keywords from the NLM data set, plus a set of additional terms, including a number of acronyms. We collected a data set of PubMed abstracts for these terms. On average, we collected 271 documents per keyword; no keyword had fewer than 125 documents, and the largest collection was 503 documents. We filtered out abstracts that were less than 15 words. We manually labeled each occurrence of each term with an identifier indicating its sense in the given context (e.g., sense 1, 2, or 3). We collected data for a total of 21 keywords. Two of these were used for training (described below), and the other 19 for our tests. Most keywords had only 2 senses in the data, with five exceptions: "BPD", "cold", "inflammation", and "nutrition" had 3 senses each, and "MCP" had four. For all five cases, the largest two senses always covered at least 70% of the examples.

For the PubMed data set, we provide two unsupervised comparison points for our system. The first is the SenseClusters system by Purandare and Pedersen (available for download at ). [9,10] Like our system, SenseClusters uses no labeled data or manual resources to make its WSD decisions. It represents the closest existing system to ours. It differs significantly from ours, however, in that it does not use the maximum-margin criterion to make its clustering decisions.

The SenseClusters system actually represents a large collection of different WSD techniques that have been developed over a number of years. In order to compare against SenseClusters, we had to select one of these techniques. The SenseClusters package has four different parameters, with between 2 and 12 values for each of them, for a total of 188 different configurations. We did not have access to any training procedures or ways of selecting values for these parameters from training data or by hand, so to be as fair as possible in our comparison, we adopted the following procedure: we ran all configurations on our test data, and chose the best-performing system to compare against. Note that this is an optimistic estimate of SenseClusters' performance, since we are in effect training the four parameters of SenseClusters on the test set.

The second unsupervised comparison point for SENSATIONAL is a common baseline that is well-known to be difficult for unsupervised WSD systems to beat, which we call the All-in-1 baseline. All-in-1 works by putting all word instances into a single cluster, and represents a system that assumes all words have only a single meaning. Its accuracy is equal to the fraction of examples that belong to the largest cluster in the true clustering.

Leroy and Rindflesch train and test their algorithm using 10-fold cross validation on the NLM data set. SENSATIONAL has one parameter, which we train on a holdout set of two keywords ("MCP" and "white") from our PubMed data set. The unknown parameter is the pruning threshold for the Backbone-Finding algorithm, and we train it using a linear search over fixed intervals. The optimal setting on this training set turned out to be a setting where the backbone stops growing if the next subtree contains fewer than one-tenth of all nodes in the graph. We use this setting for all further experiments. As we note before, we use the test data to optimize SenseClusters' four configuration parameters. SENSATIONAL and SenseClusters were both restricted to providing at most 2 clusters during testing. As we mention above, we refer to both SenseClusters and SENSATIONAL as "unsupervised" systems despite the fact that we train them on manually-labeled training data. This is because they each have a small number of parameters that could easily be set by hand, but it is more rigorous to set them using a small amount of holdout data. Referring to sysems with a small number of free parameters as "unsupervised" is common practice in Natural Language Processing literature, regardless of whether they are set by hand or with small amounts of training data [6,7].

We measure the accuracy of our unsupervised systems as follows. We first find the best possible alignment between the output clusters from the system and the clusters in the labeled data set. The "best" possible alignment is defined to be the one which results in the highest number of elements in the output clusters being aligned with a cluster in the labeled data that contains the same elements. This task is a bipartite graph-matching problem, and we use the well-known Hungarian algorithm [38] to compute the best possible matching. We then determine the accuracy as the number of elements that are correctly aligned with the labeled data set, divided by the total number of elements in the labeled data set. As is the case with any comparison of unsupervised and supervised systems, they can only be compared in terms of accuracy once the output of the unsupervised system has been aligned to the labeled test data in this manner – the unsupervised system cannot determine the correct label for each cluster, only the members of the clusters.

## Results and discussion

On the standard NLM data set, SENSATIONAL is able to outperform the baseline All-in-1 system, by 6% on average across the keywords. It does not perform as well as the supervised system by Leroy and Rindflesch, which is to be expected given that their system has access to training examples as input. However, SENSATIONAL is able to come within 5% of this supervised system without the need for a significant amount of manual input per term. Differences in accuracy between SENSATIONAL and the other two systems are statistically significant using the Chi-squared test with 1 degree of freedom. See Table 1 for details.

**Table 1:** Comparison with a supervised WSD system.

Keyword	All-in-1	L & R	SENSATIONAL
adjustment	0.62	0.57	0.56
blood pressure	0.54	0.46	0.49
degree	0.63	0.68	0.72
evaluation	0.50	0.57	0.57
growth	0.63	0.62	0.71
immunosuppression	0.59	0.63	0.59
man	0.58	0.80	0.51
mosaic	0.52	0.66	0.71
nutrition	0.45	0.48	0.42
radiation	0.61	0.72	0.65
repair	0.52	0.81	0.80
scale	0.65	0.84	0.68
sensitivity	0.49	0.70	0.74
weight	0.47	0.68	0.53
white	0.49	0.62	0.52

average	0.55	0.66	0.61

SENSATIONAL outperforms the baseline All-in-1 system by 6% on average, and comes within 5% of a supervised system for word sense disambiguation by Leroy & Rindflesch (L&R) on a standard NLM dataset. Average performance across all words is statistically significant using the Chi-square test with Yates' correction (two-tailed test with 1 degree of freedom; between All-in-1 and SENSATIONAL, Chi-square = 10.839, p = 0.0010; between SENSATIONAL and L&R, Chi-square = 7.875, p = 0.0050).

We found that SENSATIONAL requires at least 30 documents per sense in order to determine accurate clusters. Additional terms in the NLM data set did not meet this requirement, and SENSATIONAL performed below baseline on average for these terms. This is an unsurprising result, since Leroy and Rindflesch found that having fewer than 30 examples for a minority sense made it difficult for even a supervised system to find discriminating patterns in the data. Fortunately, it is much easier to add additional documents to SENSATIONAL's data set than to a supervised system's, since the data set does not need to be manually labeled. In future work, we plan to investigate more closely SENSATIONAL's accuracy as a function of the number of (unlabeled) examples it sees for each sense.

Results for our unsupervised comparison appear in Table 2. Both SenseClusters and SENSATIONAL provide statistically significant gains over the baseline All-in-1 technique, SENSATIONAL achieving an improvement of 25% over the baseline on average across the keywords. Importantly, SENSATIONAL's Max-margin technique combined with its Backbone-Finding algorithm are also able to outperform the state-of-the-art unsupervised WSD system, SenseClusters, by a statistically significant margin of 2% (two-tailed Chi-square test with 1 degree of freedom, *p *= 0.0203). The SENSATIONAL technique not only works well on average, but it also works consistently well, outperforming SenseClusters on most keywords and outperforming the All-in-1 technique on all but 2 keywords. On 8 of the keywords, it achieved a 90% accuracy or more, and on two it achieved a perfect accuracy. The data set contains a variety of ambiguous terms, and SENSATIONAL is able to perform well on all of these kinds of words, including the acronyms. These encouraging results indicate that our system may prove applicable to arbitrary biomedical terms, without the need for manual input for each term.

**Table 2:** Comparison with two unsupervised WSD systems.

Keyword	All-in-1	SenseClusters	SENSATIONAL
ANA	0.63	0.99	1.0
BPD	0.40	0.65	0.53
BSA	0.50	0.99	0.95
CML	0.55	0.99	0.90
cold	0.37	0.63	0.67
culture	0.52	0.55	0.82
discharge	0.66	0.90	0.95
fat	0.51	0.55	0.53
fluid	0.64	0.88	0.99
glucose	0.51	0.69	0.51
inflammation	0.35	0.47	0.50
inhibition	0.50	0.55	0.54
MAS	0.50	1.0	1.0
mole	0.78	0.77	0.96
nutrition	0.39	0.5	0.55
pressure	0.52	0.89	0.86
single	0.50	0.87	0.99
transport	0.51	0.52	0.57
VCR	0.79	0.65	0.64

average	0.53	0.74	0.76

SENSATIONAL outperforms the All-in-1 baseline by an impressive 23% and the SenseClusters system by 2% on a task of disambiguating terms in PubMed abstracts. Differences in performance across all words is statistically significant using the Chi-squared test with Yates' correction (two-tailed test with 1 degree of freedom; between All-in-1 and SenseClusters, Chi-square = 488.671, p < 0.0001; between SenseClusters and SENSATIONAL, Chi-square = 5.388, p = 0.0203).

## Conclusion and future work

Max-margin clustering using minimum spanning trees and our Backbone-Finding technique achieves state-of-the-art results for unsupervised word sense disambiguation without manual resources. The technique outperforms existing unsupervised techniques and comes close to the performance of a supervised technique on a variety of ambiguous biomedical terms. In addition, SENSATIONAL's clustering algorithm can run in time *O*(*N *log *N*), making it a highly attractive framework for large-scale WSD.

Thus far, we have concentrated on achieving scalability and accuracy with SENSATIONAL. Further experiments are necessary to evaluate SENSATIONAL's performance, especially how much it can improve as we add more and more unlabeled data. In future work, we plan to incorporate methods to automatically determine how many clusters exist in the data. Furthermore, we plan to incorporate existing knowledge about particular word usages from the UMLS and other databases to help disambiguate terms that already have well-defined senses.

## List of abbreviations used

UMLS: Unified Medical Language System; NIST: National Institute of Standards and Technology; WSD: Word Sense Disambiguation; NLM: National Library of Medicine; MST: minimum spanning tree; ML: Machine Learning; SVM: Support Vector Machines; JDI: Journal Descriptor Indexing; L&R: Leroy & Rindflesch.

## Competing interests

The authors declare that they have no competing interests.

## Authors' contributions

WD implemented the software and carried out the clustering experiments. MS participated in the design of the study and the creation of the test data sets. AY conceived of the project, participated in its design and coordination, and drafted the manuscript.
